# Comparative Pharmacology of Cholecystokinin Induced Activation of Cultured Vagal Afferent Neurons from Rats and Mice

**DOI:** 10.1371/journal.pone.0034755

**Published:** 2012-04-13

**Authors:** Dallas C. Kinch, James H. Peters, Steven M. Simasko

**Affiliations:** Program in Neuroscience, Department of Veterinary and Comparative Anatomy, Pharmacology, and Physiology, Washington State University, Pullman, Washington, United States of America; University of California Los Angeles, United States of America</address>

## Abstract

Cholecystokinin (CCK) facilitates the process of satiation via activation of vagal afferent neurons innervating the upper gastrointestinal tract. Recent findings indicate CCK acts on these neurons via a ruthenium red (RuR) sensitive pathway that involves members of the vanilloid (V) subfamily of transient receptor potential (TRP) channels. To further test this mechanism, the mouse provides an ideal model in which genetic tools could be applied. However, whether CCK acts by similar mechanism(s) in mice has not been determined. In the present study we explored the actions of CCK on nodose neurons isolated from Sprague Dawley (*SD*) rat and two strains of mice; *C57BL/6* and *BalbC* using fluorescence-based calcium imaging. With minor exceptions nodose neurons isolated from all species/strains behaved similarly. They all respond to brief depolarization with a large calcium transient. A significant subset of neurons responded to capsaicin (CAP), a TRPV1 agonist, although neurons from *C57BL/6* were 10-fold more sensitive to CAP than *SD* rats or *BalbC* mice, and a significantly smaller fraction of neurons from *BalbC* mice responded to CAP. CCK-8 dose-dependently activated a subpopulation of neurons with similar dose dependency, percent responders, and overlap between CCK and CAP responsiveness. In all species/strains CCK-8 induced activation was significantly attenuated (but not completely blocked) by pretreatment with the TRPV channel blocker RuR. Surprisingly, the CCK analogue JMV-180, which is reported to have pure antagonistic properties in rat but mixed agonist/antagonist properties in mice, behaved as a pure antagonist to CCK in both rat and mouse neurons. The pure antagonistic action of JMV-180 in this *in vitro* preparation suggests that prior reported differential effects of JMV-180 on satiation in rats versus mouse must be mediated by a site other than vagal afferent activation.

## Introduction

Coordination of behavioral and physiological responses following ingestion of food is critically dependent on neuronal transmission from the gastrointestinal (GI) tract to the brain [1]. The predominant sensory innervation of upper GI structures; including the stomach, duodenum, and portal vasculature, is provided by visceral afferents contained in the vagus nerve [2]. Release of the peptide cholecystokinin (CCK) from duodenal epithelium upon the arrival of nutrients into the duodenum activates vagal afferent terminals via CCK-1 receptors; a critical step in slowing gastric emptying, increasing pancreatic secretion, and facilitating the process of satiation [3]. GI projecting afferents provide key pre-absorptive nutritional information to the brain [4] and show enriched responsiveness to CCK [5]. In vagal afferents CCK acts via the low-affinity binding site [6]–[9] to decrease K^+^ [10], [11] and increase non-selective cationic conductances [11], resulting in membrane depolarization and action-potential generation [9], [12]. However, the specific cellular transduction pathway(s) and ionic conductances targeted by CCK binding at CCK-1 receptors remain incompletely characterized [13].

Over the last 25 years pancreatic acinar cells and heterologous expression systems have been used to detail the signal transduction mechanisms of the CCK-1 receptor [14], [15]. As a result we now appreciate that CCK receptor signaling is complex; with coupling to multiple G-proteins (although coupling to G_q_ is the best characterized) and activation of numerous transduction pathways (including phospholipase C (PLC), phospholipase A_2_ (PLA_2_), adenylyl cyclase, mitogen activated protein (MAP) kinase cascades, and the phosphoinositol-3-kinase (PI3K) pathway) [14], [15]. The receptor also exists in different affinity states, with each state coupling to distinct pathways and mediating specific actions [15]. While investigators have consistently found that in the rat the actions of CCK on vagal afferents are mediated by the low-affinity site [8], [16], [17], the signal pathway downstream of this activation remains elusive. For example, Heldsinger et al. (2011) reported that protein kinase C (PKC) mediates CCK-1 receptor actions via PI3K and MAP-kinase pathways; while Zhao et al. (2011) reported that inhibitors of PKC, PI3K, and PLA_2_ do not block CCK actions. The basis for these conflicting results remains unknown. Clearly multiple conductances are involved in the activation of vagal afferents [10], [11], [13], and the intracellular pathways appear complex. Zhao et al. (2011) conclude that activation is likely due to a change in phosphoinositol 4,5-bisphosphate content of the membrane which directly leads to activation of TRP conductance(s), likely to be TRPV3 and/or V4 [13].

To further test the mechanisms of CCK-induced activation of vagal afferents, the mouse would be an ideal model in which genetic tools could be applied. However, the extent to which CCK activation of vagal afferents from the mouse are similar to responses observed in the rat has not been determined. Multiple TRP channels (including TRPV1-4, TRPC1/3/5/6, TRPM8, and TRPA1) are clearly expressed in rat vagal afferents [18]; however, in select species of mouse one putative CCK mediator, TRPV3, was not detected in vagal afferents innervating the upper GI [19]. Further, the CCK-1 receptor in mouse reacts differently to the CCK analogue JMV-180. At the high affinity CCK site on the CCK-1 receptor JMV-180 behaves as a full agonist in both rats and mice, however at the low affinity site JMV-180 is a partial agonist with extremely low efficacy in the rat, often making it a functional antagonist, whereas in the mouse its efficacy is higher such that it behaves more like the full agonist CCK [20]. Because it is not fully clear how these two affinity sites couple to activation in nodose neurons and that the downstream pathways connected to CCK receptor activation in nodose neurons may be different from those characterized in acinar cells, whether CCK acts differently on mouse vagal afferents compared to the rat is an open question.

In the present study we used measurements of cytosolic calcium via fluorescent imaging to compare mechanisms of CCK activation in cultured vagal afferent neurons from Sprague-Dawley (*SD*) rats with two strains of mice (*C57BL/6* and *BalbC*). Specifically, the *C57BL/6* strain is commonly used as the background for spontaneous and targeted mutations [21]; while *BalbC* mice are used extensively to study vagally mediated neuroimmune responses in the GI tract [22]. The use of fluorescent calcium imaging enable us to directly assess the actions of added compounds on the target cells without the complication of potential indirect actions that may occur in a less reduced preparation. We found that with minor exceptions, nodose neuron from all species/strains behaved similarly. Unexpectedly, this included a pure antagonistic action of JMV-180 in both rats and mice. These findings suggest that the mechanisms of CCK induced activation of vagal afferent neurons is conserved across species, and that mouse primary vagal afferent cultures may provide a useful tool in the dissection of CCK-1 receptor signal transduction pathways. However, the existing explanation for differential behavioral effects of JMV-180 on satiation in mice versus rat, in which a mixed agonist/antagonist action versus pure antagonist action (respectively) was used to explain the differences [17], needs to be reconsidered.

## Materials and Methods

### Animals

Nodose ganglia were isolated from adult male *SD* rats (240–400 g; Simonsen Laboratories), *Balb/C* mice (20–30 g; Harlan), and *C57BL/6* mice (20–30 g; Harlan) by procedures approved by the Institutional Animal Care and Use Committee (IACUC) at Washington State University.

### Cell Isolation and Primary Culture

For all species/strains animals were always sacrificed at a similar time in the circadian cycle (∼3 hours after lights on) and measurements commenced at a similar time the following day. Nodose ganglia were isolated from animals under a deep plane of anesthesia (Ketamine, 25 mg/100 g; with Xylazine, 2.5 mg/100 g) using aseptic surgical conditions. Following a midline incision in the neck, the musculature of the next was retracted and blunt dissection techniques were used to dissociate the common vagal trunk from the carotid artery. In mice, high-magnification optics (10–100x dissecting scope; Leica Microsystems, Buffalo Grove, IL) were necessary to visualize the nodose ganglia. Once isolated, nodose ganglia were digested in Ca^2+^/Mg^2+^ free Hank’s Balanced Salt Solution containing 1 mg/mL of both Dispase II and Collagenase Type 1A (120 min at 37°C in 95% air/5% CO_2_). Neurons were dispersed by gentle trituration through silicanized pipettes, and then washed in Dulbecco’s Modified Eagle’s Medium (DMEM) supplemented with 10% fetal bovine serum (FBS) and 1% penicillin-streptomycin. Dispersed cells were plated onto poly-lysine coated coverslips and maintained in DMEM+10% FBS (37°C in 95% air/5% CO_2_). Measurements were made within 24 hours of isolation.

### Calcium Measurements

Calcium measurements were made with the fluorescent Ca^2+^ indicator Fura-2. Experiments were performed at room temperature (21°C) in a physiological saline bath (in mM: 140 NaCl; 5 KCl; 2 CaCl_2_; 1 MgCl_2_; 6 glucose; 10 HEPES with pH adjusted to 7.4 with NaOH). High K^+^ bath (HiK) had 55 mM KCl with an equimolar reduction of NaCl to 90 mM. Neurons on coverslips were loaded with 1 µM Fura-2-AM for one hour at room temperature followed by a 15 minute wash for de-esterification. Coverslips were mounted into an open chamber and constantly perfused with physiological bath. Neurons containing Fura-2 were alternately excited with 340 and 380 nm light with fluorescence monitored at 510 nm. Data points were collected at 6 second time points. Ratios of fluorescence intensity were converted to calcium concentrations using a standard curve. Data collection was controlled with MetaFluor software.

### Drugs

Drugs used are as follows (abbreviation, stock concentration, stock solvent, and supplier): cholecystokinin-octapeptide (CCK, 100 µM, H_2_O, Peptides International); Capsaicin (Cap, 10 mM, 100% EtOH, Sigma-Aldrich); Ruthenium Red (RuR, 10 mM, H_2_O, Tocris), BOC- Tyr(SO_3_H)-Nle-Gly-Trp-Nle-Asp-2-phenylethylester NH_3_ (JMV-180, 1 mM, DMSO, Research Plus Inc.).

### Statistics

For each experiment, data were collected from 2–3 nodose ganglion cell cultures from each specie/strain. Generally, protocols were designed to be within subject and analyzed using repeated measures ANOVA followed by post-hoc comparisons against control. Parameters of dose-response relationships (EC_50_, slope, maximum) were determined by sigmoid fit of the data. For antagonist studies (ruthenium red, JMV-180) all neurons received each treatment and were compared using within subject t-tests. Experiments detailing the proportion of responsive neurons (CAP, CCK, CCK/CAP overlap) report the percent responders averaged across multiple experimental days. Data are expressed as the average ± SEM. Statistical analysis was performed using SigmaStat software (Systat Software Inc., San Jose, CA).

## Results

**Figure 1:**
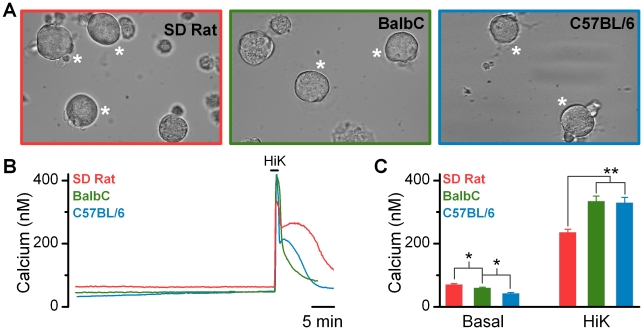
Primary cultures of nodose ganglion neurons from either rat (*SD*) or mouse (*BalbC* and *C57BL/6*) are viable and maintain similar intracellular calcium homeostasis. **A**) Brightfield photomicrographs of cultured vagal afferent neurons taken from *SD rat* (left), *BalbC* mouse (middle), and *C57BL/6* mouse (right). Viable neurons maintained a distinct cellular membrane and had smooth, rounded profiles. Measurement bar on right hand photomicrograph is applicable to all images. **B**) Representative traces of intracellular calcium concentrations from individual neurons. Brief depolarization with elevated K^+^ (HiK) produced transient increases in cytosolic calcium that decayed to baseline over time; consistent with normal calcium buffering and sequestration. **C**) Across species there was small, but statistically significant difference between basal calcium concentrations (ANOVA, P < 0.05) and Hi-K^+^ evoked transient responses (ANOVA, P < 0.01). (*SD rat*, n  =  132; *BalbC mouse*, n  =  162; and *C57BL/6 mouse*, n  =  121 neurons).

While isolated mouse nodose neurons have been previously used for electrophysiological studies [23], reports demonstrating calcium measurements in this preparation have not been made. Thus we first needed to establish the similarities/differences in behavior between nodose neurons isolated from rats versus mice. Neurons from *SD* rats versus either strain of mouse had no obvious morphological differences (Figure 1A). Cultures could be maintained for over 3 days; however, experiments reported in this communication were performed approximately 24 hrs after isolation. Like the *SD* rat, cultured vagal afferents from *BalbC* and *C57BL/6* mice were capable of maintaining low basal calcium levels for extended periods of time and exhibited rapid, robust, and reversible responses when challenged with high K^+^ (55 mM) containing baths, indicating viable expression of voltage-activated calcium channels and normal calcium sequestration pathways (Figure 1B). A small, but statistically significant, difference in basal calcium concentrations was detected across populations of neurons from the *SD* rat, *BalbC*, and *C57BL/6* mice (Figure 1B and C). In addition, a small, but statistically lower calcium response to depolarization was seen in the rat compared to mice (Figure 1C).

**Figure 2:**
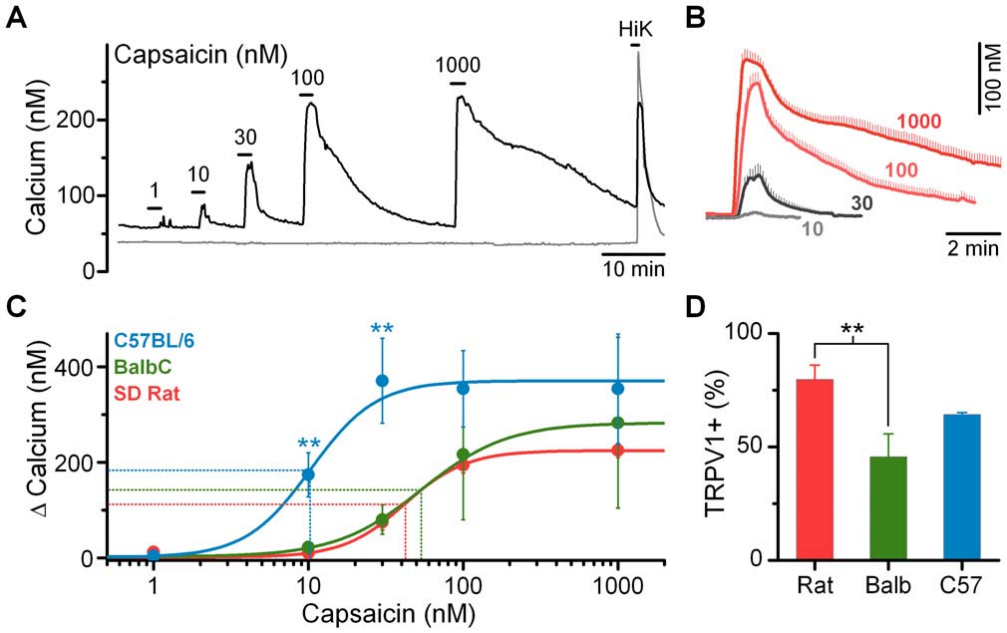
Comparison of responses to CAP in nodose ganglion neurons from *SD rat*, *BalbC mouse*, and *C57BL/6 mouse*. A) CAP induces dose dependent increases in cytosolic calcium concentrations in a subpopulation of vagal afferents (black). Some neurons are resistant to CAP even at the highest concentrations tested, but increase calcium following depolarization indicating normal cell viability (gray). Traces are from representative neurons taken from a *SD rat*. **B**) High concentrations of CAP increased both the peak and integrated calcium responses. Traces are averages ± SEM across responsive neurons from *SD rat* (n  =  22). **C**) CAP dose response relationships across species: *C57BL/6 mouse*, n  =  8, EC_50_  =  10 ± 2 nM CAP, slope  =  2.09 ± 0.53, max  =  371 ± 89 nM calcium; *BalbC mouse*, n  =  4, EC_50_  =  54 ± 26 nM CAP, slope  =  1.59 ± 0.70 max  =  283 ± 179 nM calcium; and *SD rat*, n  =  22, EC_50_  =  42 ± 6 nM CAP, slope  =  2.21 ± 0.29, max  =  225 ± 15 nM calcium. Peak calcium influx was not significantly different between groups (ANOVA, P  =  0.84); however, the EC_50_ was lower in neurons from *C57BL/6* mice compared to both *SD* rat and *BalbC* mice due to larger calcium responses at the 10 nM (P < 0.001) and 30 nM (P < 0.001) CAP concentrations. **D**) Percent of neurons responsive to CAP (100 nM) relative to all HiK responsive neurons within an isolation (*SD rat*, 80 ± 6%, combined n  =  51/63; *BalbC*, 46 ± 10%, combined n  =  38/82; *C57BL/6*, 64 ± 1%, combined n  =  27/42). Both strains of mice had lower percentages of CAP responsive afferents; with the *BalbC* (Holm-Sidak post-hoc test, P  =  0.009), but not the *C57BL/6* (Holm-Sidak post-hoc test, P  =  0.40) afferents statistically lower compared to those taken from the *SD* rat.

Primary vagal afferent neurons are broadly classified as A-, Aδ-, or C-fibers based on the extent of myelination and resulting axonal conduction velocities [24]; with extensively myelinated A-fibers able to conduct action-potentials ten to twenty-fold faster than unmyelinated C-fibers. The TRPV1 ion channel is preferentially expressed in C- and Aδ-fibers and not in A-fibers; providing a pharmacological marker of fiber subtype in cultured neurons where myelination is not maintained [25]. The TRPV1 agonist, CAP, dose-dependently activated a subpopulation of vagal afferents identifying them as TRPV1-expressing C- and Aδ-fibers (Figure 2A). Across TRPV1+ afferents, increasing concentrations of CAP increased both the peak magnitude and duration of the calcium transient (Figure 2B). The threshold to maximal response in all species/strains occurred over an approximately ten-fold concentration range (Figure 2C). The magnitude of the calcium response was not significantly different between species/strains (Figure 2C); however, the EC_50_ for CAP was lower in afferents taken from *C57BL/6* mice (Figure 2C). The proportion of CAP sensitive afferents from the *SD* rat was 80 ± 6%; somewhat higher than previously reported values [26]. Both strains of mice had lower percentages of CAP responsive afferents; with the percent responsive in *BalbC*, but not the *C57BL/6*, reaching statistical significance (Figure 2D).

**Figure 3:**
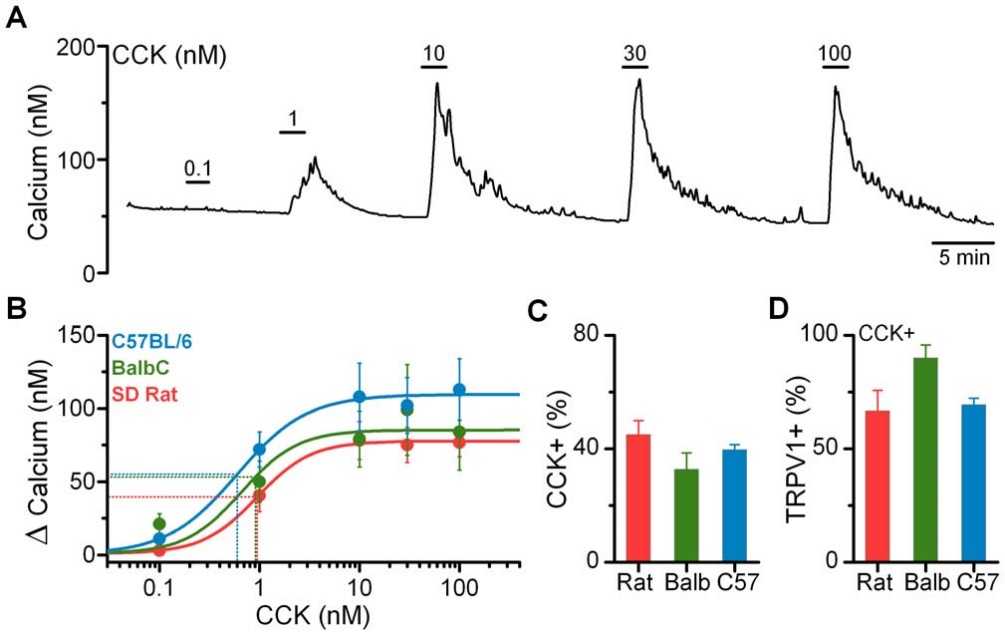
Cholecystokinin (CCK) produces nearly identical activation of cultured vagal afferents from the rat and mouse species. A) CCK produces dose dependent increases in cytosolic calcium concentrations in a subpopulation of vagal afferents. Neurons were also challenged with CAP to determine afferent subtype and HiK to verify viability (data not shown). Trace is from a representative neuron taken from a *C57BL/6 mouse*. **B**) CCK dose response relationships across species/strains: *C57BL/6 mouse*, n  =  4, EC_50_  =  0.60 ± 0.22 nM CCK, slope  =  1.24 ± 0.26, max  =  108 ± 23 nM calcium; *BalbC mouse*, n  =  5, EC_50_  =  0.92 ± 0.77 nM CCK, slope  =  3.03 ± 29.5, max  =  99 ± 31 nM calcium; and *SD rat*, n  =  7, EC_50_  =  0.95 ± 0.35 nM CCK, slope  =  1.58 ± 0.40, max  =  77 ± 10 nM calcium. **C**) Percent of total viable neurons tested responsive to CCK. Results from challenges with 10 nM CCK were used to determine population characteristics (*SD rat*, 45 ± 5%, combined n  =  35/75; *BalbC*, 33 ± 6%, combined n  =  41/111; *C57BL/6*, 40 ± 2%, combined n  =  38/90). **D**) Percent of CCK responsive neurons also activated by CAP (*SD rat*, 67 ± 9%, combined n  =  15/21; *BalbC*, 90 ± 6%, combined n  =  17/19; *C57BL/6*, 69 ± 3%, combined n  =  13/19).

In the rat, CCK activates a subpopulation of vagal afferent neurons via binding at the CCK-1 receptor subtype [6]–[8]. CCK produces a rapid calcium transient that requires the influx of extracellular calcium [7], [8] independent of voltage-gated calcium channels [13] and that begins to fall even with the continued presence of CCK. As in the rat, CCK increased cytosolic calcium concentrations in a subgroup of neurons from both the *C57BL/6* and *BalbC* mice (Figure 3). CCK-induced activation was dose-dependent with maximal activation occurring by 10 nM CCK (Figure 3A and 3B). Across species/strains the dose-response relationships were nearly identical (Figure 3B). In this sample, CCK activated approximately 40% of all nodose neurons across species/strains (Figure 3C); consistent with previous findings in the rat [8]. A majority of CCK responsive neurons were also sensitive to CAP (Figure 3D); suggesting preferential activation of C- and Aδ-fibers by CCK [12]. There were no significant differences in the magnitude and dose response characteristics of CCK induced activation between CAP-sensitive and CAP-insensitive afferents (data not shown). Because of this the responses to CCK from both CAP-sensitive and CAP-insensitive neurons were combined for the summarized dose-response curves and calculated overall response rates.

**Figure 4:**
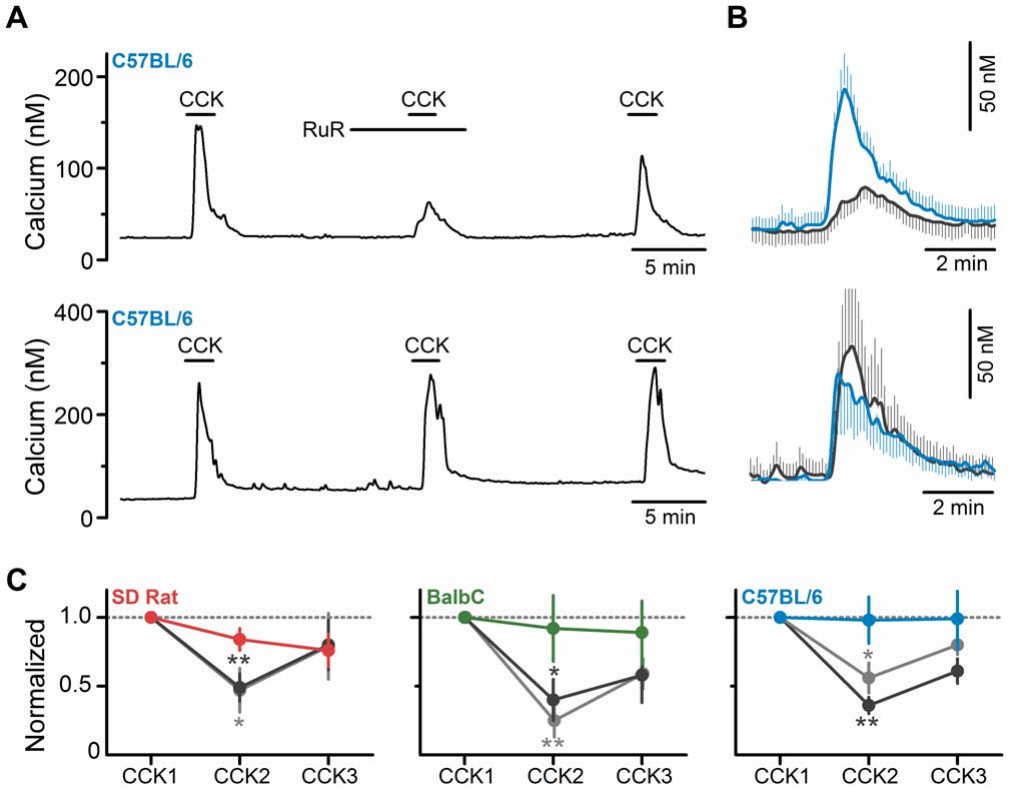
CCK activation of primary vagal afferents is dependent on a Ruthenium Red sensitive conductance. A, upper: representative trace showing attenuation of CCK (10 nM) induced increase in cytosolic calcium following pretreatment with RuR (10 µM) in a nodose neuron from a *C57BL/6 mouse*. **Bottom:** control response to three consecutive challenges showing neither attenuation nor desensitization of the CCK induced response with repeated challenges. **B, upper:** Across neurons, RuR (10 µM) attenuated the average CCK activated calcium transient, while the control (**bottom**) was unaltered. Traces are the average ± SEM of responsive neurons (RuR, n  =  6, black line; control n  =  6, colored line) **C)** RuR significantly reduced CCK induced calcium influx in the *SD rat* (**left**; control (red line and points), n  =  4; RuR 10 µM (grey), *p < 0.05, n  =  8; and RuR 30 µM (black), **p < 0.01, n  =  6), in the *BalbC mouse* (**middle**; control (green line and points), n  =  5; RuR 10 µM (grey), **p < 0.01, n  =  9; and RuR 30 µM (black), *p < 0.05, n  =  5), and in the *C57BL/6 mouse* (**right**; control (blue line and points), n  =  6; RuR 10 µM (grey), *p < 0.05, n  =  6; and RuR 30 µM (black), **p < 0.01, n  =  9). Values are normalized to the first CCK challenge (CCK1) and are expressed as average ± SEM.

In rat CCK activates vagal afferent neurons in large part by increasing a RuR-sensitive conductance; likely a TRPV channel subtype [13]. We confirmed this action in rat (Figure 4C, left hand panel). Further, we found that RuR had similar actions in neurons from both strains of mice (Figure 4). By itself (10 µM or 30 µM), RuR produced no change in the basal calcium concentration, but significantly attenuated the CCK-induced calcium transient (Figure 4A and B, top panels). In control experiments, repeated exposure to CCK did not produce a significant desensitization (Figure 4A and B, bottom panels). In all species/strains reduction in the calcium transient induced by CCK by 10 µM and 30 µM RuR were similar in magnitude although recovery tended to be more complete after treatment with 10 µM. These findings demonstrate that the linkage between CCK receptor activation and calcium influx through a RuR-sensitive conductance(s) in nodose neurons is preserved between rat and mice. However, given the inability of RuR to completely block the response, additional RuR-insensitive pathways are likely to also be involved.

**Figure 5:**
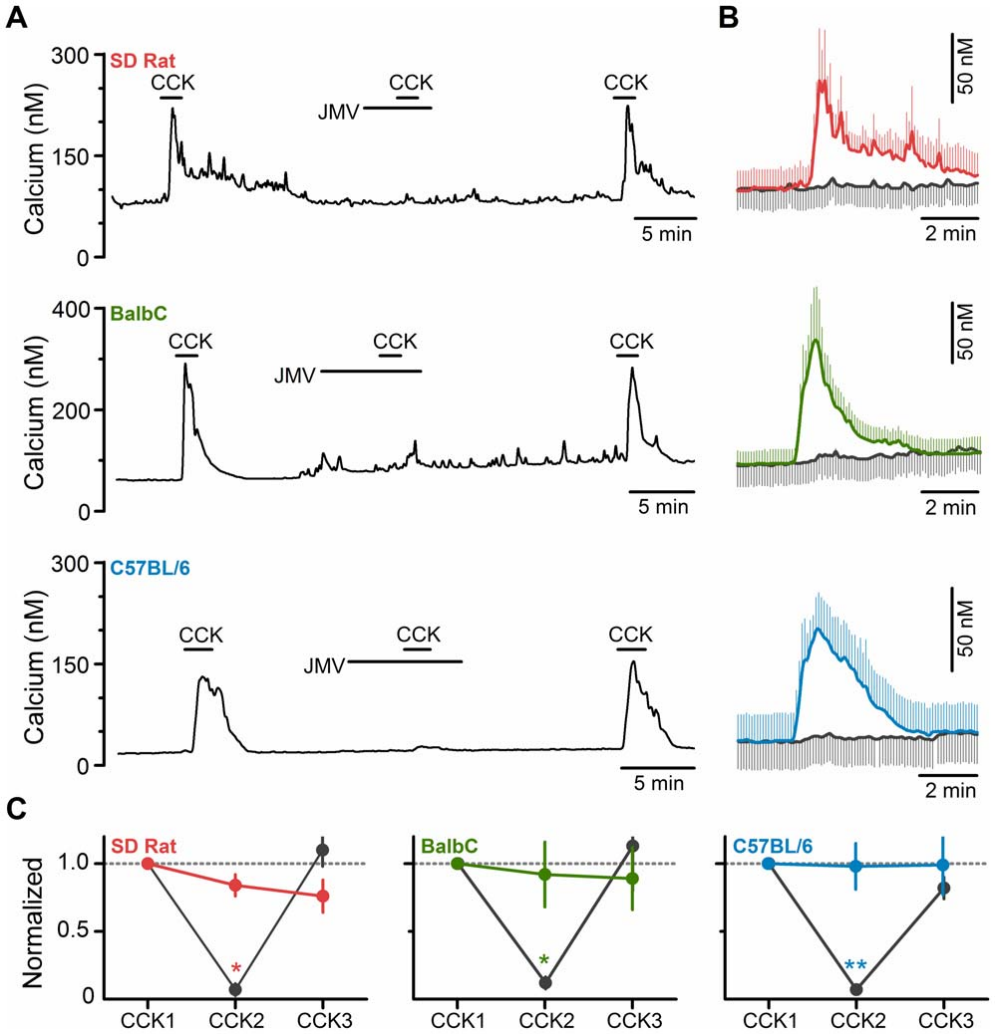
CCK activation of primary vagal afferents is blocked by CCK analog JMV-180 (JMV) across species. A) Representative traces showing effect of JMV-180 (100 nM) on the calcium response induced by CCK (10 nM) in *SD rat* (top), *BalbC mouse* (middle), and *C57BL/6 mouse* (bottom). **B**) Average calcium levels in response to challenge with CCK alone (colored, first challenge) and challenge to CCK in presence of JMV-180 (grey). Traces are the average ± SEM of responsive neurons (*SD rat*, n  =  4; *BalbC mouse*, n  =  5; *C57BL/6 mouse*, n  =  6). **C**) JMV significantly decreased CCK-induced calcium influx in a reversible manner. Colored points and lines are from three challenges to CCK without JMV present in the middle challenge. Black points and lines are when JMV-180 was present during the second challenge with CCK (CCK2). Results have been normalized to the first CCK challenge (CCK1) and are the average ± SEM. (*SD rat*, p < 0.05, n  =  4; *BalbC*, p < 0.05, n  =  5; *C57BL/6*, p < 0.01, n  =  6; *p< 0.05 and **p<0.01 by paired t-test).

CCK-1 receptors have both high (pM) and low (nM) affinity ligand binding sites that mediate distinct signaling cascades and physiologic processes [27]. The CCK analogue, JMV-180, is a full agonist at the high affinity site, but is a partial agonist at the low affinity site [28] with different degrees of efficacy in the rat versus the mouse [29], [30]. Thus if the specific response under study is mediated by the low affinity site, JMV-180 can have primarily agonistic actions (often observed in the mouse), or in the presence of CCK, to have antagonistic actions, especially in the rat [28]–[30]. In rat nodose neurons we found that JMV-180 by itself had no effect to induce a calcium signal (100 pM – 100 nM, data not shown), and that it produced a near complete block of the actions of CCK in a reversible manner (Figure 5), thus confirming the low affinity site as the relevant site for CCK actions in this preparation and that the weak partial agonism reported for JMV-180 at this site is inadequate to induce a calcium response in nodose neurons. Unexpectedly, we found that JMV-180 behaved identically in both strains of mice as it did in the rat. It had no actions by itself (100 pM to 100 nM, data not shown), and it produced a near complete block of the actions of CCK in a reversible manner (Figure 5).

## Discussion

The primary finding of this study is the conserved distribution and cellular mechanism of CCK activation at vagal afferent neurons across rat and mouse species. In the rat, CCK activates vagal afferents through the CCK-1 receptor isoform at low nanomolar concentrations, consistent with binding at the low affinity site [8], [16], [17]. This concentration-response relationship is maintained in mouse cultured vagal afferents, with activation resulting in a monophasic rise in cytosolic calcium which begins to desensitize in the continued presence of CCK. The overall percentage of CCK responsive neurons, and their distribution across afferent phenotype (A- vs. C- & Aδ-fibers), as functionally defined with CAP, were not different between species/strains. Furthermore, the response to CCK in all species/strains was equally attenuated by RuR. Unexpectedly, JMV-180 had pure antagonistic actions in the mouse when prior studies on pancreatic acinar cells would have suggested we should have observed some agonistic actions in this preparation [20], [29], [31]–[33]. These observations demonstrate that while vagal afferents isolated from the mouse may be a useful tool for further dissecting CCK actions on nodose neurons, one cannot assume that the relationship between activation at a particular affinity state/site and activation of subsequent downstream pathways as characterized in the acinar cells will simply transfer to the downstream pathways activated by CCK-1 receptors in nodose neurons.

### CAP Identification of Vagal Afferent Phenotypes

Reductions in food intake by CCK are initiated via activation of CAP sensitive subdiaphragmatic vagal afferents [34]. CAP is a selective agonist at the TRPV1 ion channel and serves to operationally define unmyelinated (C-fiber) and lightly myelinated (Aδ-fiber) afferents in the absence of conduction velocity measurements [25]. In all species/strains tested, increasing concentrations of CAP increase both the peak and duration of the cytosolic calcium transient in a similar manner; identifying subpopulations as expressing TRPV1. We found the dose-response relationship for CAP to be very steep (Hill coefficients ∼2). This steep dose-response relationship is similar to prior reports for both rat [35], [36] and mouse [37]. Interestingly, the dose-response curve was shifted significantly to the left in afferents from *C57BL/6* mice compared to either the *SD* rat or *BalbC* mouse. That there is a difference between mouse strains, when the primary structure of TRPV1 is similar, suggests a contextual co-factor present in one but not the other strain may influence sensitivity to CAP. A second difference noted was that the percentage of CAP responsive vagal afferents was significantly lower in the *BalbC* mouse versus the *SD rat*, with the *C57BL/6 mouse* having an intermediate number. It remains unknown whether this finding corresponds to a differential distribution of myelinated versus unmyelinated fibers *in vivo,* and if there are functional consequences to having lower percentages of TRPV1-expressing vagal afferents. Such a difference would predict lower TRPV1-mediated glutamate release at the central terminals of vagal afferents in the *Balb/C mouse* [38], [39], which may impact the relay of satiation signals via the vagus.

### Conserved CCK Activation of Vagal Afferents Across Species

CCK directly activates a subpopulation of vagal afferent neurons through binding at the CCK-1 receptor [6]–[8]. In the rat CCK consistently activates ∼40% of the neurons obtained from measurements in which the innervation targets of nodose neurons are unidentified [8], [40]. We have previously found that when retrogradely transported dyes are used to identify the stomach or duodenum as the innervation target, ∼70% of the nodose neurons respond to CCK [5]. Given that ∼70% of the afferent vagal fibers innervate GI structures [41], one can calculate that if a neuron responds to CCK, it is highly likely to be a neuron that innervates a GI structure (70% of 70% equals ∼50% of all fibers). This is actually a slight overestimate of how many unidentified nodose neurons should respond to CCK, which could be explained by the assumption that even within GI innervating vagal afferent fibers, there is a selective responsiveness to CCK in neurons that innervate only the stomach and/or duodenum. Since we did not verified that a similar enrichment of CCK responsive fibers also occurs in nodose neurons in the mouse, we do not know for certain that a similar restriction on the innervation targets for CCK responsive neurons is also present, but given the similarities in other factors (percentage responsive to CCK and CAP, and overlap between CCK and CAP responsive neurons), one is led to a similar conclusion that responsiveness to CCK by a nodose neuron from a mouse indicates a high likelihood that the neuron innervated a GI structure. The findings that the CCK and CAP response patterns are so similar in mouse and rat is consistent with *in vivo* studies investigating CCK induced reduction of food intake in mice [42], and suggest conserved mechanisms of CCK activation of vagal afferents between rats and mice.

### Identifying the Membrane Conductance(s) Activated by CCK

CCK acting at vagal afferent neurons produces a net depolarizing current resulting in increased membrane excitability [11], [12]. This activation occurs independently of voltage-activated calcium currents (N-, P/Q-, L-, and T-type) [13]; but relies on extracellular calcium influx [8]; suggesting a member of the TRP channel family [13]. Our previous work in the rat found that the inorganic dye ruthenium red (RuR), a broad spectrum TPRV ion channel pore blocker, largely attenuated CCK-induced increases in calcium [13]. Presently, we report that CCK-induced increases in cytosolic calcium are equally sensitive to RuR in both mouse species relative to the rat. This finding suggests the CCK-targeted conductance(s) may be the same across species. Considering CCK activates vagal afferents in the presence of the TRPV1 antagonist SB366791 and in CAP insensitive afferents, alternative TRPV channels (TRPV2–6) are more likely potential mediators. It is noteworthy that the attenuation of the calcium signal by RuR was incomplete in all species/strains suggesting that while a RuR-sensitive conductance has a prominent role in the response to CCK, there are likely to be additional pathways for calcium entry that are activated.

### Actions of the CCK Analogue JMV-180

The CCK receptor couples to multiple signaling pathways through distinct affinity states on the CCK-1 receptor [15], [43]. Analogues of the CCK molecule have been developed previously and enabled pharmacological dissection of CCK/CCK-1 receptor binding, activation and signal transductions pathways in pancreatic acinar cells [44]–[46]. The truncated CCK-8 analogue, JMV-180, is proposed to have differential effects at the high and low affinity binding sites on CCK-1 receptors. In both rat and mouse acini it is an agonist at the high affinity site which mediates amylase secretion, but an antagonist at the low affinity site only in the rat [47]. The high affinity site is thought to stimulate acini cells through PLA_2_ [48], [49] independently of the PLC/diacylglycerol (DAG)/inositol trisphosphate (IP_3_) pathway [33], [50], [51]. In contrast, the low affinity site couples to activation of the PLC/DAG/IP_3_ pathway [28], [33]; where low levels of activation augment amylase secretion, but high levels of activation suppress amylase secretion [33]. This dichotomy in signaling likely underlies the differential effects of JMV-180 between the rat and mouse species. JMV-180 is a partial agonist at this low affinity site with different degrees of efficacy in the mouse versus the rat [20], [29], [31]–[33]. For example, in the mouse JMV-180 is 45% as effective as CCK in stimulating phosphoinositol (PI) breakdown, whereas in the rat it is only 20% as effective [29]. Because JMV-180 does not cause inhibition of amylase secretion in the rat but does in the mouse [20], [33], [47] it has been discussed as being an agonist at this site in the mouse but an antagonist in the rat; whereas at events more proximal to the activated receptor, such as phosphoinositides breakdown (Bianchi et al., 1994), the partial agonist behavior of the analogue is more apparent.

Since the low affinity site mediates CCK-induced vagal afferent activation [8], [16], we expected JMV-180 would block CCK action in rat and mimic them in the mouse strains. However, we found it produced no effect by itself and completely blocked CCK actions in both species. The low affinity site on the CCK-1 receptor couples through a G_q_ mediated pathway to activate PLC and subsequent generation of DAG and IP_3_ [28], [33]. Further, since CCK-responses are sensitive to RuR and inhibited by the PLC inhibitor, U73122, but not sensitive to PKC inhibitors, we previously concluded that PLC induced changes in the membrane phospholipid content leads to increased activation of a RuR-sensitive TRP channel [18], [52]. Our current results suggest that either CCK operates via another yet identified pathway from the PLC/DAG/IP_3_ activation pathway to activate vagal afferents; or that the limited efficacy of JMV-180 to activate this pathway [29] is inadequate to induce activation of RuR-sensitive TRP channels. Thus although JMV-180 is frequently referred to as an agonist at the low affinity site in the mouse, because it is only a partial agonist at this site with limited efficacy, in the case of activating vagal afferents from the mouse, it is a functional antagonist.

In regards to satiation, JMV-180 has been reported to block CCK-induced satiation in rat, but to promote satiation in the mouse, an effect blocked by CCK-1 receptor antagonism [17]. Our results demonstrate JMV-180 has only antagonistic actions on isolated mouse nodose neurons, and suggest the satiating effect of JMV-180 in mice is not mediated by vagal afferent activation. It is possible that this discrepancy may be due to strain differences in mice tested since the previous *in vivo* work used outbred ICR mice (Harlan Sprague Dawley) [17]. In acini the partial agonist behavior of JMV-180 is influenced by both primary structure and the cellular context in which the receptor is expressed [53]–[55]. Thus it is possible that JMV-180 works differently in outbred ICR mice than in *Balb/C* or *C57BL/6 mouse* models. Alternatively, the very high doses of JMV-180 administered to observe satiation may have been sufficient to activate CCK receptors at multiple sites, producing a net satiating effect. Resolution of this discrepancy may prove difficult *in vivo* considering the distribution of CCK-1 receptors in tissues controlling digestion and food intake [56].

### Conclusions

The findings in the current study indicate the general phenotype of CCK activation of vagal afferent neurons is conserved between rat and mouse species. As such, genetic knockouts, and other experimental manipulations in the mouse, may allow for the identification of the cellular transduction pathway and membrane conductance(s) targeted by CCK. Further, detailed mechanisms of CCK activity delineated in the mouse are likely to generalize to the rat. Of specific note is that resolving the basis for the lack of action of JMV-180 in isolated nodose neurons from mouse relative to its known actions in acinar cells may prove insightful into the final delineation of the transduction pathway activated by CCK in nodose neurons. Continued progress utilizing these animal models should clarify CCK induced actions at vagal afferent neurons and its role in feeding and neuroimmune responses.
